# Optimising care coordination strategies for physical activity referral scheme patients by Australian health professionals

**DOI:** 10.1371/journal.pone.0270408

**Published:** 2022-07-14

**Authors:** Francis A. Albert, Aduli E. O. Malau-Aduli, Melissa J. Crowe, Bunmi S. Malau-Aduli

**Affiliations:** 1 College of Medicine and Dentistry, James Cook University, Townsville, QLD, Australia; 2 College of Public Health, Medical and Veterinary Sciences, James Cook University, Townsville, QLD, Australia; 3 Division of Tropical Health and Medicine, James Cook University, Townsville, QLD, Australia; University of Glasgow MRC/CSO Social and Public Health Sciences Unit, UNITED KINGDOM

## Abstract

Physical activity (PA) has been identified as an essential tool for the prevention and management of multi-morbidity in patients. Coordination of patients’ care through interventions like physical activity referral schemes (PARS) could foster the utilization of PA. This study explored the views of General Practitioners (GPs) and Exercise Physiologists (EPs) as key stakeholders, for optimizing patient care and efficiency of PARS. Sequential explanatory mixed methods design was used to explore the perceptions of these health professionals on PA and coordination strategies for PARS patient care. Data analyses included descriptive and inferential statistics for questionnaires and theoretical framework analysis for the semi-structured interviews. Participants demonstrated a good knowledge of PA and valued PARS. However, the findings unravelled external factors, inter-organisational mechanisms, and relational coordination obstacles that hinder efficient coordination of PARS patient care and delay/limit beneficial health outcomes for patients. Incentivising the PARS initiative and empowering patients to seek referral into the programme, are strategies that could boost PARS efficiency. Improving inter-professional relationships between GPs and EPs could lead to enhanced PARS functionality and efficient coordination of care for patients with chronic diseases.

## Introduction

Globally, chronic diseases are the leading risk factors for disability and mortality [1]. Three out of every five deaths are attributed to chronic conditions including cardiovascular disease, cancer, chronic lung disease and diabetes [2]. Research has linked numerous chronic diseases to the lack or shortage of physical activity (PA) and urged its promotion [3–5]. To enhance PA, myriads of integrated care programmes, including physical activity referral schemes (PARS) that support and promote PA to patients through interprofessional collaboration among health professionals, have been developed in various settings across the world [6–8]. In Australia, one of such pathways is the Medicare-funded chronic disease management (CDM) plan, where patients with chronic diseases can access rebates for five sessions per year with any allied health professionals (AHPs) of their choice, including exercise physiologists (EPs) [9]. Australian patients need a formal GP referral to access these rebates and would have to pay out of pocket or a combination of out-of-pocket cost and private health insurance for subsequent sessions if they exhaust their rebatable sessions within a year [10]. Over 90% of Australians see a GP at least once a year [11], and about half of these patients have multimorbidity [12]. Leveraging on GPs’ accessibility to patients and complimenting it with the expertise of PA specialists like EPs could help reduce the rising cases of chronic and complex disease conditions [13].

However, current evidence calls into question the effectiveness of care coordination between health professionals. This is ascribable to time constraints, lack of knowledge, shared understanding of common goals and role clarity, cost implications and weak collaborations influenced by organisational culture and structure [14–18]. For example, studies on the coordination of care for patients have shown that the stewardship of some health professionals such as GPs is essential [19]. However, these doctors may not feel obliged to coordinate patient care or be part of the health care team [20]. Similar issues could be hindering the functionality of PARS, considering that this programme is a typical example of coordinated care. For instance, studies have highlighted that including stakeholders like GPs in the design and development of PARS initiatives [21] and supporting them to promote the programme [22] are critical to PARS success. Nonetheless, other studies have revealed that crucial decisions are taken by stakeholders involved in care coordination without inputs from GPs [23]. This suggests that health professionals’ coordination of care for PARS participants warrants further exploration. Seeking health professionals’ perspectives could aid amelioration of the identified bottlenecks in the structure and process of PARS, foster patients’ health outcomes and the optimisation of inter-professional care coordination strategies.

Coordination of care through interprofessional collaboration could be enhanced by adopting care frameworks focused on the promotion of teamwork, interprofessional channels and fostering the health and wellbeing of the populace [24, 25]. A detailed assessment of care coordination frameworks led to the adoption of an emergent care coordination framework [26]. This framework pinpoints critical components of care coordination that promote responsiveness, service consolidation, and expertise for improved patient health outcomes [26]. The model (S1 Appendix) proposes that links between the functionality of healthcare interventions like PARS and key care coordination variables influence patients’ health outcomes. The framework was employed in this study to aid in-depth understanding of the GPs and EPs’ experience of care coordination for PARS patients and their perceived areas of contention in the coordinated care process.

Therefore, this mixed methods study employed an emerging care coordination framework to investigate the perceptions of Australian health professionals (GPs and EPs) regarding the coordination of care for PARS participants to determine effective ways to enhance the programme’s efficacy.

The study sought to answer the following research questions (RQs):

RQ1. What are Australian health professionals’ knowledge, beliefs, and attitudes towards PA and PARS?
RQ2. What are Australian health professionals’ views regarding the coordination of PARS care for participants and how to optimize the programme?

## Methods

### Study design

A sequential explanatory mixed methods design guided by a pragmatic approach [27] that included two study phases was used to answer the research questions. A general overview of the experiences (knowledge, beliefs, and attitude) of health professionals (GPs and exercise physiologists) in coordinating PARS care for patients was investigated in the first (quantitative) phase of the study. In the second phase (qualitative), semi-structured interviews were conducted to understand participants’ perception about care coordination through PARS. The findings from the quantitative phase of the study guided the development of the qualitative interview protocol and selection of participants for the qualitative phase.

Mixed methods design involves collecting, analysing and integrating of quantitative and qualitative data within the same study to answer specific research questions [27]. Combining both methods in a single study and triangulation of findings aided comprehensive and critical analysis of health professionals’ account of the complicated issues surrounding the coordination of patients’ care via PARS [27].

The ethical clearance for this study was secured from the Human Research Ethics Committee (HREC) of James Cook University (JCU) (Reference number: H7661). Designated health organisation representatives who assisted with participant recruitment were provided with the ethics approval details. Participants were further provided with the relevant information sheet, their privacy rights, and the possible benefits of the study. While keeping confidentiality and anonymity, electronic and verbal consents were sought from participants before the commencement of both phases of the study [28].

### Quantitative phase

The first phase of the study answered RQ1 and utilised quantitative data collection and analytical techniques to examine GPs and exercise physiologists’ (EPs) knowledge, beliefs and attitudes about PA and PARS. An *a priori* G-Power analysis [29] revealed that 64 participants per group was required to achieve 80% power for detecting a medium-sized effect at a 0.05 level of statistical significance.

### Survey development

A cross-sectional survey design was used to collect data from participants in this phase of the study. Issues identified from past PARS and care coordination studies informed the development and structure of the survey tool [16, 30]. The questionnaire was subdivided into five sections: participant demographics, knowledge, beliefs, attitudes and recommendations for improved PA and PARS. Each section featured different types of questions including a 5-point Likert scale type (ranging from “1 = Strongly disagree” to “5 = Strongly agree”) for the belief section, multiple-choice and dichotomous questions for the knowledge, PA behaviour and recommendation sections. For the dichotomous knowledge of PA questions, each correct answer had a score of one (1), while a wrong answer had zero. Key stakeholders in PA and PARS including health professionals served as a review panel and verified the survey’s content validity. The survey was pilot-tested on 15 randomly selected participants (10 EPs and 5 GPs) and the feedback was used to revise the survey items.

### Data collection

Data were collected electronically via Survey Monkey® (by SVMK Inc.) between November 2019 and August 2020. Eligible participants were GP or EP, above 18 years and registered to practice in Australia. Participants were recruited via their work affiliations (organisation or clinical settings where the GP or EP worked) and online fora including Twitter and Facebook. While GPs were recruited from clinical settings across Australia, EPs were mainly recruited via Exercise and Sports Science Australia (ESSA), the professional body for EPs. The first named author (FAA) facilitated and handled the correspondence for the recruitment application process. The process included the application and provision of an online survey link to participants or their affiliations. There was also an option of hard copies. To increase survey responses, reminder emails and incentives (a chance to win one of 10 $100 or five $200 gift vouchers) were used. Participants were assigned pseudonyms to protect their identity and were asked an optional question to request their participation in an interview.

### Quantitative data analysis

Data management and analysis were performed using IBM’s SPSS statistics software version 26. The survey data (including the pilot-tested data) were analysed using descriptive statistics (for the demographic, PA behaviour and recommendation data), independent T-Test (for knowledge data) and Mann-Whitney U (for the belief data) statistical tests. Participants were categorised into groups (GP and EP groups) based on their professional affiliation. Results were displayed as frequencies and means ± SD and a p-value of ≤0.05 was considered significant.

### Qualitative phase

Participants who agreed to participate in the qualitative phase were purposively selected (selecting a heterogeneous mix of respondents based on their survey responses, demographics, and availability to inform a greater understanding of the coordination of care in PARS referrals) to provide responses to RQ2. Semi-structured open-ended questions were then used to interview eligible participants between September and December 2020.

### Qualitative data collection

A draft interview guide with open-ended questions based on the findings from the first stage of the study was pilot tested with five (5) participants (three EPs and two GPs) by the first author (FAA) and transcripts checked by another author (BSMA) to confirm the credibility and suitability of the questions. The findings from the pilot test were used to refine the interview guide (S2 Appendix). Telephone interviews were used to explore participants’ perceptions on coordination of care through PARS. Each interview commenced with a verbal acknowledgement of consent. Interviews continued until data saturation was achieved [31].

Ten (10) semi-structured interview questions were used to explore participants’ views about coordinating PARS care for patients who utilized the programme’s services. Interview questions explored participants’ perception of their roles in coordinating PARS referrals for patients, PARS knowledge, beliefs and attitudes, influences of other health professionals (GPs or EPs), perceived challenges and benefits of PARS and their thoughts on how to improve the effectiveness of the patient care coordination for PARS. Prompts and probes were developed concerning the interview topics, when necessary, to kindle further responses from participants. Telephone interviews lasted between 16 and 50 minutes.

The interviewer (FAA) concluded each interview with a summary of interview accounts to secure trustworthiness and mutual understanding between both parties [32]. Data saturation was reached at the 22^nd^ interview after which three more interviews were conducted, totalling 25 interviews. Pseudonyms were assigned to respondents to aid anonymity.

### Qualitative data analysis

Before data analysis, interviews were audio taped, transcribed verbatim and identity information removed. Interview transcripts (including those from the pilot test) were imported into QSR International’s NVivo version 12 for theoretical framework analysis. Framework analysis involves the screening, sorting, and charting of data based on crucial issues and themes [33]. The identified themes were then deductively mapped to the care coordination framework. Framework analysis was employed to help identify the factors that influence health professionals’ coordination of PARS care and their perception of the programme’s effectiveness. It involves a five-step process: (1) Reading and re-reading the textual data to familiarise with the data, (2) Identifying, devising, or refining a thematic framework to facilitate data analysis, (3) Indexing the data to corresponding themes, (4) Charting the identified themes (5) Mapping and interpreting the themes generated [34]. Information source triangulation, member checking, review and resolution of disconfirming evidence and researcher verification were used to secure the trustworthiness of the findings [35]. Two researchers (FAA and BSMA) independently coded the data and developed and mapped all themes against those of the care coordination model. Discrepancies regarding the addition, removal or refinement of codes and themes were resolved in a consensus meeting with all research team members. The protocols of the consolidated criteria for reporting qualitative research (COREQ) checklist helped guide the qualitative phase (S3 Appendix) [36].

### Triangulation of quantitative and qualitative data

Framework analysis [34] and the principles described by O’Cathain et al. [37] facilitated the triangulation of the findings from both phases of this study. The procedure involved (1) independently, analysing the findings and developing threads (themes) from each phase of the study, (2) linking the threads from the first to the second phase of the study, so that they could be interpreted together and (3) drawing overarching conclusions and meta-inferences by integration and refining the findings from both phases of the study [26, 38, 39].

## Results

Two hundred and thirty-eight (238) participants (121 GPs and 117 EPs) responded to the surveys. Thirty-one (31) incomplete survey responses (19 from GPs and 12 from exercise physiologist—EPs) were excluded, while 207 completed responses (including 105 from EPs and 102 from GPs) were analysed. This response rate exceeds the required 64 participants per group required from the statistical power analysis.

### Quantitative phase

Table 1 portrays the demographic characteristics of the two study participant groups, including age, gender, location, years of experience and the types of patients referred or received in PARS referrals. Overall, there was an approximately equal representation of male (52%) and female (48%) respondents, with Queensland recording more participants than any other state across Australia. The EPs were younger in age (28–37 years) compared to the GPs (39%) who were mostly above 38 years of age. More participants lived in cities (43%) compared to regional (40%) and rural (17%) centres. EPs (37%) reported between two (2) to five (5) years of working experience, while GPs (39%) recorded above five (5) years of working experience. GPs and EPs identified similar patient conditions in relation to the top four reasons for referral (overweight/obese, diabetes, cardiovascular diseases and musculo-skeletal disorders). For GPs, the ranking order was overweight/obese (85%), diabetes (80%), cardiovascular diseases (79%) and musculo-skeletal disorders (64%), indicating that they mostly referred overweight or obese patients. For EPs, the ranking order was musculo-skeletal disorders (82%), overweight/obese (74%), diabetes (72%) and cardiovascular diseases (70%), indicating that they admitted more patients with musculoskeletal disorders (data not shown). For both groups, the least referred or admitted into PARS were older or frail patients (data not shown).

**Table 1:** Descriptive characteristics of participants (GPs and EPs) (N = 207).

	GPs	EPs	Total (%)
**Health professionals**	N (%) 102 (100)	N (%) 105 (100)	207 (100)
**Age (years)**			
≤ 27	11 (11)	40 (38)	51 (24)
28–37	31 (30)	45 (43)	76 (37)
≥ 38	60 (59)	20 (19)	80 (39)
**Gender**			
Male	59 (58)	48 (46)	107 (52)
Female	43 (42)	57 (54)	100 (48)
**State/Territory**			
Queensland	61 (60)	40 (38)	101 (49)
Victoria	18 (18)	24 (23)	42 (20)
New South Wales	2 (2)	21 (20)	23 (11)
South Australia	16 (16)	2 (2)	18 (9)
Western Australia	1 (1)	12 (11)	13 (6)
Other States/Territories (Australian Capital Territory, Tasmania, and Northern Territory)	4 (3)	6 (6)	10 (5)
**Environment (Location)**			
Capital city	48 (47)	40 (38)	88 (43)
Regional	42 (41)	41 (39)	83 (40)
Rural	12 (12)	24 (23)	36 (17)
**Years of experience (years)**			
< 2	44 (44)	32 (31)	76 (37)
2–5	11 (11)	39 (37)	50 (24)
> 5	46 (45)	34 (32)	80 (39)

### PA/PARS attitudes

As portrayed in Table 2, most participants (91%) indicated that they were involved in PA. A further assessment showed that 99% of the EPs were involved in PA, compared to 82% of the GPs. Additionally, 48% of the GPs reported being either inactive or below 150 minutes of PA per week, compared to only 1% of the EPs. Notwithstanding, the 1% inactive EP could be an artifact because the option “Not active” recorded a 0% score for EPs when asked about their exercise intensity. The three most common reasons participants gave for taking part in PA included healthy and lifestyle benefits (24%), to relieve stress (19%) and enjoyment (18%). EPs who considered PA as a hobby (55%), means of socialization (51%) or example to patients (46%) more than doubled the GPs (20%, 17% and 12%, respectively). Also, while 45% of GPs indicated that they took part in PA for weight-loss reasons, only 28% of EPs endorsed this point. Participants indicated that they referred (77% of GPs) or received (91% of EPs) patients via PARS. Pursuing this further in the method for patient referral, however indicated that over one third (32%) of these referrals were initiated by patients themselves (one in every three patients). Forty-four per cent (44%) of GPs indicated that they don’t receive feedback from EPs on the patients they refer to them while the majority of EPs (91%) claimed the opposite (i.e., they provide feedback to GPs on the patients they received from them).

**Table 2:** Participants’ PA/PARS attitudes (N = 207).

	GPs N (%)	EPs N (%)	Total (%)
**Are you involved in PA?**			
Yes	84 (82)	104 (99)	188 (91)
No	18 (18)	1 (1)	19 (9)
**Minutes of PA per week**			
Not active	13 (13)	1 (1)	14 (7)
< 150	35 (35)	9 (9)	44 (22)
150–299	31 (31)	40 (38)	71 (35)
≥ 300	21 (21)	55 (52)	76 (37)
**Intensity of PA**			
Not active (≤ 1.5 METs)	13 (13)	0 (0)	13 (6)
Low (1.6–2.9 METs)	9 (9)	2 (2)	11 (5)
Moderate (3.0–5.9 METs)	55 (54)	43 (41)	98 (48)
Vigorous (≥ 6.0 METs)	25 (25)	59 (57)	84 (41)
**Reason(s) for taking part in PA**			
Healthy lifestyle benefits	79 (78)	99 (94)	178 (24)
Relieve stress	54 (53)	88 (84)	142 (19)
Enjoyment	46 (45)	91 (87)	137 (18)
Hobby	20 (20)	57 (55)	77 (10)
Weight loss	46 (45)	29 (28)	75 (10)
Socialize	17 (17)	53 (51)	70 (9)
Example to patients	12 (12)	48 (46)	60 (8)
Skill development/competition	0 (0.0)	9 (9)	9 (1)
**Patient referral via PARS**			
Yes	78 (77)	95 (91)	173 (84)
No	23 (23)	10 (9)	33 (16)
**Method of patient referral**			
GP initiated (Referral letter)	52 (51)	63 (66)	115 (58)
Patient initiated	41 (41)	23 (24)	64 (32)
Within practice referrals	9 (8)	10 (10)	19 (10)
**Feedback to GPs on PARS intervention**			
Yes	56 (56)	88 (91)	144 (73)
No	44 (44)	9 (9)	53 (27)

### PA knowledge

As shown in Table 3, independent-samples t-test revealed a significant difference between the PA knowledge scores of GPs (80 ± 15.5) and EPs (90 ± 11.9), *t* (157) = -5.4, *p* <0.001, two-tailed). The magnitude of the difference in the means (mean difference = -3.70, 95% *CI*: -5.05 to -2.36) was moderate (eta squared = 0.13).

**Table 3:** Participants’ PA knowledge (N = 207).

	GPs	EPs	p-values
**Knowledge Questions**	(N = 102) % correct	(N = 105) % correct	
Physical activity is any movement that involves contraction of muscles?	75	92	<0.001*
Physical activity has to be high intensity to benefit health?	82	97	<0.001*
Climbing the stairs is a form of physical activity?	94	100	0.011*
Exercise is form of physical activity	95	97	0.445
Physical activity is only beneficial if performed for at least 20 minutes at a time?	84	97	0.001*
The recommended PA for adults is at least 150 minutes low–moderate physical activity per week or 10, 000 steps per day?	79	81	0.781
Adults are encouraged to engage in 30 minutes of physical activity per week or 5000 steps per day to confer relevant health benefits?	52	68	0.022*
**Total percent score ± SD**	80 ± 15.5	90 ± 11.9	0.0001*

*p <0.05

### PA and PARS beliefs

Generally, EPs reported slightly stronger beliefs in PA and PARS value than GPs (Table 4). When participants were asked if they were confident in their ability to prescribe PA, 67% of GPs either strongly agreed or agreed, while 99% of EPs either strongly agreed or agreed. A Mann-Whitney U test was calculated for all the total scores for the belief questions to determine the difference in the levels of belief between GPs and EPs. The results indicated that EPs are more agreeable with the statements about the value of PA counselling to their field of practice, health professionals’ confidence in prescribing PA and PA benefits to their patients. GPs (Md = 14, n = 102) and EPs (Md = 20, n = 105) with a large effect size (*r = 0*.*85)*, U = 141.000, *z* = -12.289, *p* = .0001.

**Table 4:** Participants’ PA and PARS beliefs (N = 207).

	GPs	EPs	
**Belief Questions**	N (%)	N (%)	Combined Mean score (SD)
*Physical activity counselling is important in my field of practice*			*4*.*18 (0*.*76)*
Strongly agree	61 (57)	81 (77)	
Agree	40 (39)	22 (21)	
Neutral	1 (1)	2 (2)	
Disagree	0 (0)	0 (0)	
Strongly disagree	0 (0)	0 (0)	
**Mean group score (SD)**	**3.59 (0.51)**	**4.75 (0.48)**	
*I am confident in prescribing PA to my patients*			*3*.*92 (1*.*26)*
Strongly agree	32 (31)	91 (87)	
Agree	37 (36)	13 (12)	
Neutral	24 (23)	0 (0)	
Disagree	7 (7)	0 (0)	
Strongly disagree	2 (3)	1 (1)	
**Mean group score (SD)**	**2.88 (0.99**)	**4.84 (0.50)**	
*PA is beneficial to my patients*			*3*.*44 (0*.*83)*
Strongly agree	69 (68)	98 (93)	
Agree	32 (31)	7 (7)	
Neutral	1 (1)	0 (0)	
Disagree	0 (0)	0 (0)	
Strongly disagree	0 (0)	0 (0)	
**Mean group score (SD)**	**3.67 (0.49)**	**4.93 (0.25)**	

*p =* 0.0001

### Perceived benefits, barriers and recommendations about PARS

As displayed in Table 5, GPs and EPs identified similar reasons in their responses to the pre-set answers on their perceptions of the benefits of PARS (Patient-reported improved health outcome, presence of objectively measured output and reduced the work burden placed on doctors/GPs) respectively. For barriers, while most EPs (79%) saw the lack of knowledge on referral pathways as the main hindrance to the programme’s functionality, more GPs (50%) noted the scarcity of PARS as the main barrier. Again, while 55% of EPs viewed the lack of patient motivation to take up PARS as a key barrier, only 5% of GPs supported this statement. For recommendations, GPs indicated improved visibility of EPs whilst more EPs indicated ongoing interactions between GPs and EPs to improve referral programs.

**Table 5:** Perceived benefits, barriers, and recommendations about PARS (N = 207).

		GPs N (%)	EPs N (%)	Total (%)
**Benefits**	Patient-reported improved health outcome (improved health condition due to PA programme)	76 (75)	93 (90)	169 (42)
	Presence of objectively measured outcome (The health gains can be measured)	53 (53)	76 (74)	129 (32)
	Reduces the work burden placed on doctors/GPs	44 (44)	62 (60)	106 (26)
**Barriers**	Lack of knowledge on referral pathways	37 (36)	81 (79)	118 (19)
	Physical activity support services are highly undervalued	41 (40)	69 (67)	110 (18)
	Scarcity of referral pathways	51 (50)	38 (38)	89 (14)
	Inadequate consultation time	22 (22)	47 (46)	69 (11)
	Lack of financial incentive	35 (34)	32 (31)	67 (11)
	Patients not motivated to take up PARS referral	5 (5)	57 (55)	62 (10)
	Lack of national collective goal or coordination process on referral pathways	20 (20)	42 (41)	62 (10)
	Lack of reference materials	14 (14)	28 (27)	42 (7)
**Recommendations**	Ongoing interactions between GPs and EPs	66 (65)	96 (93)	162 (23)
	Improved visibility of EPs	73 (72)	87 (84)	160 (23)
	Education about referral pathways	37 (37)	68 (66)	105 (15)
	An overview of available referral pathways	43 (43)	49 (48)	92 (13)
	Easily accessible or ease of use of PARS	47 (46)	42 (41)	89 (13)
	Simplify PARS documentation process (documentation should be optimised for disease management)	20 (20)	29 (28)	49 (7)
	Financial incentives or subsidies for patients	8 (8)	34 (33)	42 (6)

### Qualitative phase

Twenty-five (25) participants eight (8) GPs (32%) and 17 EPs (68%) participated in the individual telephone interviews. Participants included 14 males (56%) and 11 (44%) females. Qualitative findings were mapped unto the constructs of the care coordination theoretical framework. Based on the care coordination constructs, five overarching themes (1) External factors, (2) Patient knowledge and motivation, (3) (Inter)organizational mechanisms, (4) Relational coordination and (5) Outcomes were identified. Each theme is discussed below, and the representative quotes are presented in Table 6.

**Table 6:** Triangulation of study findings embedded within the care coordination framework.

Care Coordination factors (Overarching theme)	Quantitative findings	HCP Quotes		Synthesis of Findings
		*GPs*	*EPs*	
**External Factors** These included limited government support, increased burden of cost (extra sessions) and poor continuity of care for patients	Undervaluing of physical activity support services was the second most highlighted barrier to PARS effectiveness by the participants (40% GPs and 67% EPs). Participants (44%) recommended a review of available referral pathways and 20% proposed giving financial incentives or subsidies to patients to enhance the functionality of PARS.	GPs voiced their discontent with the limited number of EPC sessions allocated to patients *“Interventions from allied health professionals is not a one off*. *Take the exercise physiology for instance*, *there’s a need first of all to assess the patient*, *which may be done at the first visit and develop a plan of intervention and then you now need to begin to implement that and then there’s a need to monitor see how it is*. *And this cannot be done with just five visits and sometimes not even the entire five because the patient wants to also use some of it for some other allied health professionals*, *so no*. *Five is certainly not enough” (Dr ON 52)*	EPs perceived that the government undervalued their services. They also reported that the free EPC sessions were inadequate and impacted on continuity of care. *“One of the barriers is just that the government severely underestimates our worth and just not pay enough in terms of the Medicare rebate” (AN 31)* *“The main problem with only having a couple of sessions would be that we don’t get that continuity of checking up on the client” (JT 26)*	Improving PARS incentives (e.g., financial incentives) for HCPs could motivate stakeholders to promote PARS and enhance the programme’s functionality. Increasing the CDM rebates or sessions for patients could foster PARS uptake by patients and enhance the programme’s functionality.
**Patient Knowledge and Motivation** Participants’ perceptions about PARS showed that motivating patients and providing adequate knowledge regarding PARS are essential for effective uptake of the programme	While 55% of EPs viewed the lack of patient motivation to take up PARS as a critical barrier, only 5% of GPs supported this point. Additionally, More EPs (79%) indicated the lack of knowledge on referral pathways among patients as a major barrier to the uptake and effectiveness of the PARS programme in comparison to GPs (36%) Participants (77% of GPs and 91% of EPs) indicated that they referred or received patients via PARS. Pursuing this further in the method for patient referral, however indicated that over one third (32%) of these referrals were initiated by patients themselves.	GPs reported that their discussion with patients is guided by patients’ interests. *“Patient wise*, *they may not be interested in that discussion at that point in time because they may have come for a different concern*. Dr CF 43 *“The patients are happy especially if the patient education has occurred at the time of diagnosis*. *At the time of diagnosis*, *the background education helps a patient to comprehend what they need and how the exercise physiologist will be key or will be part of their management team*. *So*, *they are quite happy to go” (Dr CL 44)*	EPs indicated that patient are the ones providing information about PARS to GPs to seek for referral into the programme. EPs were dissatisfied with how GPs’ leave crucial PARS referral decisions to patients *“Most of the time*, *if the patient is going to get referred by their GP*, *is because they ask for it*. *And most of my experience with that*, *isn’t that necessarily that the GPs has instigated it” (NK 29)* *“I found the last few years a lot of GPs just say to their client; oh*, *go and find an EP and then I would refer you*. *So*, *GPs are getting a bit lazy by saying to the patient*, *you go and find them*, *and I’ll refer you” (MD 43)*	Empowering patients to decide on their referral choices or delegating a designated HCP such as a nurse might coordinate the referral of patients into PARS and enhance uptake, the referral process and reduce the burden of work on GPs. Insights on effective ways to promote PA and PARS initiatives to patients prior to taking up the programme’s initiative could foster uptake and enhance the efficiency of the programme.
**(Inter)organisational Mechanism** Major (inter)organisational obstacle to the success of the PARS programme included poor EP accessibility, knowledge gaps, complicated administrative processes and time constraints	Participants (GPs = 50% and EPs = 38%) highlighted the scarcity of PARS as one of the barriers to the functionality of the programme An exploration of participants’ location showed a similar distribution of HCPs across capital cities (43%) followed by regional areas (40%) and less in rural areas (17%). Most EPs (79%) saw the lack of knowledge on referral pathways as the main barrier to the programme’s functionality, while more GPs (51%) noted the scarcity of PARS as a barrier.	GPs regarded the scarcity of EPs and burdensome administrative processes as critical factors that impede the usability of the PARS programme. *“The availability of EPs in the first place*. *Compared to other allied health fields EPs are still few and far between*. *There is concentration of EPs only in urban areas*, *most of the regional areas have no EP whatsoever and even urban areas they are not that readily available*. *So*, *availability of the EPs is certainly an issue” (Dr ON 52)* *“We will start with knowledge*. *So*, *like I said*, *that most patients would be with their conditions for a long period of time*. *Which means that one way or the other doctors have not identified that someone else could be involved in that treatment*. *So*, *there’s that knowledge gap it is still there” (Dr CL 44)* *“The amount of paperwork involved in setting up the care plan*, *the team care arrangement and the referral and then doctors not having enough time for a longer consult or to take the patients questions and all that” (Dr GE 44)* *“We are restrained as GPs*, *because you’ve got fifteen minutes to deal with*. *I mean*, *not even coming for any concerns relating to exercise*, *but we use that opportunistically*, *especially for somebody who is overweight*, *has a chronic condition*. *So*, *it is something you just [briefly] discuss but well most times most GPs don’t have that time” (Dr CF 43)*	EPs echoed the opinions of the GPs and attributed it to GPs’ time constraints and minimal information sharing opportunities. They specified that the information deficiency might be around the value of the services they provide to patients. *“I’m the only EP in say a 10K radius*. *So*, *I suppose some of the barriers are*, *the doctors just don’t know who to contact” (SU 33)* *“With GPs referring I think it can be a lack of knowledge about the benefits that we can provide*. *And the safety that we can guarantee for these people with education*. *So that’s not always known*, *and I think that creates barrier” (LR 28)* *“If you give them your information by the end of the day*, *I find they are just so busy*. *They don’t have the time to actually think about when they have seen a patient who would benefit from seeing an exercise physiologist” (MD 43)*	Promotion of PARS initiatives, better remuneration under CDM and incentivising the services of EPs could attract more HCPs into the profession and increase their availability and accessibility. Making PARS information more accessible to patients and key HCPs like GPs through workshops and constant reminders and printed materials like pamphlets could foster PA and PARS knowledge and increase the programme’s usability.
**Relational Coordination** Participants ‘perceptions about the relationship between EPs and GPs bothered around EP roles, exchange of information, quality of the interprofessional collaboration and sharing of common goals	Overall, EPs recorded a slightly stronger belief in the value of PA and PARS compared to GPs. Forty-four per cent (44%) of GPs indicated that they don’t receive feedback from EPs on the patients they refer to the EPs, while the majority of EPs (91%) claimed the opposite (providing feedback to GPs on the patients they refer to them). In relation to recommendation, GPs (72%). emphasised EPs’ improved visibility while EPs (93%) emphasised ongoing interactions between GPs and EPs.	GPs admitted that they lacked understanding of the roles of EPs but were in favour of interprofessional coordination of care. *“Now*, *sometimes there’s a struggle with respect to*, *this is my opinion anyway; what are the boundaries of a physiotherapist and an exercise physiologist*. *If there is a major difference as to when to involve a physiotherapist and when to involve an exercise physiologist (Dr CF 43)* *“I try to base my judgement not just on the feedback from the exercise physiologists*, *I also base my judgement on how well the patient had fared by engaging with their service*.*” (Dr KC 42)* *“Every patient with a chronic disease condition requires multidisciplinary approach to the management*. *The GP will be at the centre of it to coordinate and make the necessary referrals*, *coordinate the treatments*, *receive reports from the allied health professionals and review the patients as we go on” (Dr GE 44)*	EPs said that GPs exhibited a lack of knowledge about EP duties and were also too busy, which hindered access to PARS for patients. *“There’s a big gap in GP understanding of EPs role and what they could do*. *I think a lot of people miss out on the service just because the GPs aren’t referring” (AD 32)* “*What I gather though when you send those [report] to the surgery*, *they are just filed automatically by reception staffs*. *The GPs don’t get to see them unless the patient goes back and they say*, *oh let’s see how you went*. *But they don’t*, *so if the patient doesn’t go back or the conditions get sorted*, *so they don’t need to talk about that again*. *I think often the GP doesn’t see those letters” (SU 33)* *“If a GP refers someone to an EP and that client gets great outcomes from that EP*. *They are going to trust that GP*, *they going to keep going back to that GP*, *and you know*, *whenever there’s an issue—it’s a nice little loop*. *That’s how it should be*, *we should be looking out for each other and having the client’s outcomes as our first and foremost goal” (ER 26)*	The lack of clarity on the roles of EPs among GPs could be leading to wrong referrals, this could be addressed, through education and training workshops. Professional ongoing interaction among HCPs through seminars and workshops and in foundational training could foster the knowledge of the roles of EPs and provide insights on the value and scope of their services. Information sharing among HCPs is key to the success of PARS and needs improvement. The ability for HCPs to freely share professional information among themselves could promote access to PARS, speed up the process and enhance its ease of use and effectiveness.
**Outcome** Participants commended the PARS programme and indicated that it had enhanced patients’ health outcomes. Need for improvement on team and inter-organisational outcomes	Participants (EPs– 82% and GPs—62%) reported objectively measured improved patient health outcomes as a major benefit of PARS.	GPs found PARS to be helpful in helping users achieve their health goals and regained the ability to perform their usual activities. *“I’ve had patients who have had knee surgeries*, *… So*, *I refer them to exercise physiologist*, *and then after a while*, *they were back on their feet and back to their sporting activities” (Dr KC 42)* *“It’s very important because the more we engage with exercise physiologists*, *the better for the community especially*, *for GPs who are in remote areas where people seldom engage in exercises*. *You know*, *it is very good that they refer their clients to exercise physiologists” (Dr KC 42)*	EPs saw value in PARS’ ability to help clients perform certain activities and daily chores with ease. However, they harped on the delayed referral of patients to PARS and how this could make it difficult for the clients to achieve their goals. *“I’ve had patients say that they can do the gardening or mowing without getting out of breath probably tired*. *They’ve got the confidence to get back on a normal road bike again*. *They walked to work” (NK 29)* *“We used to only see people when they were all done*, *and all the damage is already done and trying to rebuild the person from ashes is hard experience” (LR 28)*	Participants perceived PARS to be invaluable in helping patients achieve their health outcomes. Improved collaboration among HCPs such as GPs and EPs and timely referral of patients into PARS could enhance the programme’s viability, functionality, and better health outcome for patients.

#### External factors

Participants highlighted the effects of some external factors which serve as obstacles to the effectiveness of the PARS programme. These obstacles included limited government support in terms of inadequate Medicare-funded CDM sessions which ultimately led to increased burden of cost (extra sessions) for patients. All participants perceived that PARS was undervalued by the government due to the few free CDM-funded sessions and Medicare rebates allocated to patients. The inadequate funding of PARS served as a barrier to the programme’s uptake and effectiveness. EPs reported that the limited funding for the programme compelled them to charge extra fees to compensate for the time they invest in patient care. They perceived that the undervaluing of their services ultimately impacts on continuity of care for patients who are unable to afford ongoing engagement with the PARS programme. GPs supported the EPs’ notion and indicated that the current five Medicare-funded sessions patients get to see any allied health professional of their choice are not enough and should be reviewed.

#### Patient knowledge and motivation

An investigation of participants’ perceptions about PARS showed that motivating patients and providing adequate knowledge regarding PARS are essential for effective uptake of the programme. GPs expressed the importance of providing background PA and PARS knowledge to patients to help the patients appreciate and value the services of EPs, with subsequent better motivation and uptake of the referral. EPs substantiated the views of the GPs. Nonetheless, the EPs indicated that the patients were more knowledgeable than the GPs about PARS and often the patients were the ones providing information about PARS to GPs and proactively seeking referral into the programme.

#### (Inter)organisational mechanism

The participants expressed strong beliefs in the value of PARS and the need to coordinate care for patients through the programme. GPs spoke of the importance and need to collaborate with other HCPs. EPs substantiated the views of the GPs and emphasised the value patients attach to the involvement of GPs who help them achieve their health and wellness goals. However, poor visibility of EPs was identified by GPs as a major obstacle to the success of the PARS programme. They regarded the scarcity of EPs, particularly in regional and remote settings, as a critical factor that impedes the usability of the PARS programme. The limited availability was also reiterated by EPs who indicated that EP to patient ratio was low. Both GPs and EPs highlighted knowledge gap as another major obstacle to the success of the programme and this was attributed to poor information sharing about the benefits it has to offer. EPs also indicated that being time-poor, and overburdened with work, GPs might struggle to promote PARS to patients even if they have the information. In response to this, GPs faulted the PARS documentation process and time constraints as limiting factors for promoting PARS to patients.

#### Relational coordination

GPs and EPs had different approaches to patient care in relation to PARS. While GPs proposed PA and PARS interventions to patients and leave patients with the choice of uptake, EPs emphasised the importance of motivating patients and guiding them to see the benefits of taking up the intervention. This could be partly attributed to the lack of understanding of the roles and capabilities of EPs and how this affects patients’ ability to access the PARS programme. EPs were of the opinion that GPs were mostly unaware of the services that EPs offer. GPs admitted that they lacked understanding of the roles of EPs and perceived a need to clarify the boundary in the roles of EPs and other AHPs such as physiotherapists. Both participant groups indicated that an improved interprofessional relationship could be beneficial in the coordination of optimum care for patients. They stressed the need for feedback and information sharing to foster trust and improved functionality of the PARS programme. GPs indicated that they don’t receive feedback from EPs on the patients they refer to the EPs, while the EPs claimed that the GPs were not proactive enough in following up with the feedback from the PARS consultation. Instead, the feedback is often filed away by administrative staff, and this might prevent information from getting across to the doctors.

#### Outcome

Both participant groups reiterated the value of PARS in helping users achieve their health goals and regain the ability to perform their usual activities. GPs viewed collaboration with EPs as very essential and crucial to the improved wellbeing of the patients. The EPs emphasised the invaluable impact of shared experiences among PARS members. However, they expressed concerns about the delayed referral of patients to PARS and how this could make it difficult for the clients to achieve their health goals.

### Triangulation/Integration of findings

The findings from both the quantitative and qualitative phases were synthesised and mapped to the themes of the care coordination framework. Table 6 portrays a summary of the integrated findings and representative participant quotes.

## Discussion

This mixed methods study employed a care coordination framework to explore the perceptions of key PARS stakeholders (GPs and EPs) regarding the coordination of care for PARS patients to determine effective and sustainable ways of fostering the health outcomes of patients and enhancing the effectiveness of PARS. Quantitative findings highlighted that GPs and EPs have good knowledge of PA and value PARS. Qualitative findings unravelled external factors, inter-organisational mechanisms, and relational coordination obstacles that hinder the ability to efficiently coordinate PARS patient care and delay/limit beneficial health outcomes for patients. These results substantiate our previous findings on the perspectives from patients [40] and uncover the need for policies that would reflect value for PARS initiatives, promote information sharing and strengthen inter-professional relationships between GPs and EPs [16, 41]. Similarly, a mixed methods study by Buckley et al. [22] highlighted that a multifaceted approach is required to support GPs in promoting PA and PARS programmes.

An assessment of the external factors influencing the coordination of care by participants in the quantitative phase showed that barriers, including an undervaluing of the PARS programme and lack of financial incentive hinder the ability of GPs and EPs to coordinate patient care. A study by Clark et al. [42] revealed that health professionals including doctors are hindered from implementing PA guidelines by the lack of insights on referral options, programme resources, increasing workload and the absence of incentives. These findings were substantiated in the interview when respondents lamented about the poor funding and low rebatable sessions. Thus, supporting PARS stakeholders with incentives (e.g., increased EPC for patients and increased funding for health professionals) could enhance the programme’s uptake, functionality and boost health outcomes for patients [43, 44].

Participants gave dissenting views on how the characteristics of patients influence the way they coordinate care. In the surveys, while GPs and EPs indicated strong beliefs in the value of PA for managing chronic conditions and praised the impacts of the PARS programme, they disagreed on the enthusiasm of patients to take up PARS initiatives. The interviews revealed that GPs and EPs value background PA and PARS education for patients before referral into PARS. EPs however, perceived that GPs were abdicating their frontline roles as PARS gatekeepers leading to patients initiating PARS referrals. A mixed methods study that explored the effects of empowering patients through self-management support, concluded that collaboration between patients and healthcare providers, access to self-management information and more diversified care for chronic diseases could optimise patient empowerment [45]. Therefore, enhanced information sharing among stakeholders and patients, promoting the benefits of PARS and empowering patients to take up PARS intervention could foster adherence to programme interventions and optimal health outcomes for patients [10, 40].

Examining the inter(organisational) mechanisms and relational coordination among health professionals revealed a complex coordination of care PARS process. Enhanced functionality of PARS would require further insights into the roles of EPs and improved accessibility to their services [18, 42]. These issues could be addressed through ongoing professional interaction among health professionals such as GPs and EPs, particularly during the foundational training years to become a healthcare professional and in-service training via workshops or seminars [46]. An exploration of the views of exercise referral trainers regarding the uptake and attendance in PARS highlighted that those who deliver the programme could benefit from ongoing training and support from colleagues [47]. In addition, raising PARS awareness through different sources such as the media and printed materials like pamphlets could augment the programme’s insight, accessibility and functionality [16]. Participants were full of praise for the positive outcomes that have come out of the PARS programme. GPs and EPs commended the impacts of the PARS initiatives for helping to foster the health and wellbeing of patients, enhancing the bonding among community dwellers, and reducing the burden on the healthcare system. Therefore, developing strategies that would aid PA promotion and PARS initiatives could foster collaboration among health professionals and help them coordinate the best care for patients, share information efficiently, and achieve sustainable goals [13, 48].

To the best of our knowledge, this is the first study in an Australian context, that has used a care coordination model to explore the experiences of GPs and EPs regarding PARS. As key PARS stakeholders, the inputs of GPs and EPs would strengthen the evidence base on the coordination of care for PARS participants. Employing a sequential explanatory mixed methods approach ensured integration and in-depth understanding of the findings. However, the findings should be cautiously interpreted in the light of the following limitations: (1) The study considered only the perceptions of Australian GPs and EPs. (2) Although using a random sampling strategy facilitated the collection of information that could be useful for successful implementation of care coordination goals among health professionals, this strategy could have biased the responses of health professionals, as some respondents with affinity for PA and PARS could have been attracted to the study. (3) Finally, the results from this study were based on self-reported opinions of participants, which could have been either over- or under-estimated, owing to the specialty of health professionals who took part in the study.

Health professionals’ views about care coordination for PARS participants have revealed desired outcomes. However, obstacles in most of the critical factors (external factors, patient knowledge and motivation, (inter)organisational mechanisms and relational coordination) facilitating the functionality of the care pathway limit the programme’s efficiency. Therefore, strategies that would promote GPs and EPs’ behavioural change towards effective care coordination are needed to foster quality care for patients, improve their health outcomes and forge a solid and efficient healthcare system.

## Conclusion

This study set out to critically appraise the views of GPs and EPs on the coordination of PARS care for patients to improve its efficiency and actively inform policy on PARS development or restructuring. Participants displayed good knowledge and firm belief in PARS, but health professionals, particularly GPs, require more knowledge, support, and incentives to promote, drive and coordinate PARS initiatives for patients effectively. Strategies to foster inter-professional relationships and efficient exchange of information between GPs and EPs are urgently required. This would enable insights into the roles and boundaries of PA specialists like EPs and unearth the values of the services they render. The findings from this research could inform policies that will enhance interest in PARS utilisation by frontline health professionals like GPs and the coordination of optimum care for patients, particularly those with multi-morbidity. A policy shift towards improving current incentives such as better PARS pay for health professionals and increased free EPC visits for patients could enhance positive mindsets and attitudes towards PARS initiatives among stakeholders. A broader view of all key PARS stakeholders, including GPs, EPs, and patients, concerning efficient ways to coordinate care for PARS participants could be invaluable to the initiative’s success.

## Supporting information

S1 Appendix Emergent care coordination framework. (TIFF)

S2 Appendix Interview guide for participants (GPs and exercise physiologists—EPs). (PDF)

S3 Appendix The consolidated criteria for reporting qualitative research (COREQ) checklist. (PDF)

## List of abbreviations

CDM: Chronic Disease Management
CMD: College of Medicine and Dentistry
EPs: Exercise Physiologists
ERS: Exercise Referral Scheme
EPC: Enhanced Primary Care
GPs: General Practitioners
HREC: Human Research Ethics Committee
JCU: James Cook University
PA: Physical Activity
PARS: Physical Activity Referral Scheme
RQs: Research Questions
