# Supplementary figures and images for: Optimising care coordination strategies for physical activity referral scheme patients by Australian health professionals
Source: PLoS One. 2022 Jul 14;17(7):e0270408. doi: 10.1371/journal.pone.0270408 (PMC9282539; doi:10.1371/journal.pone.0270408)

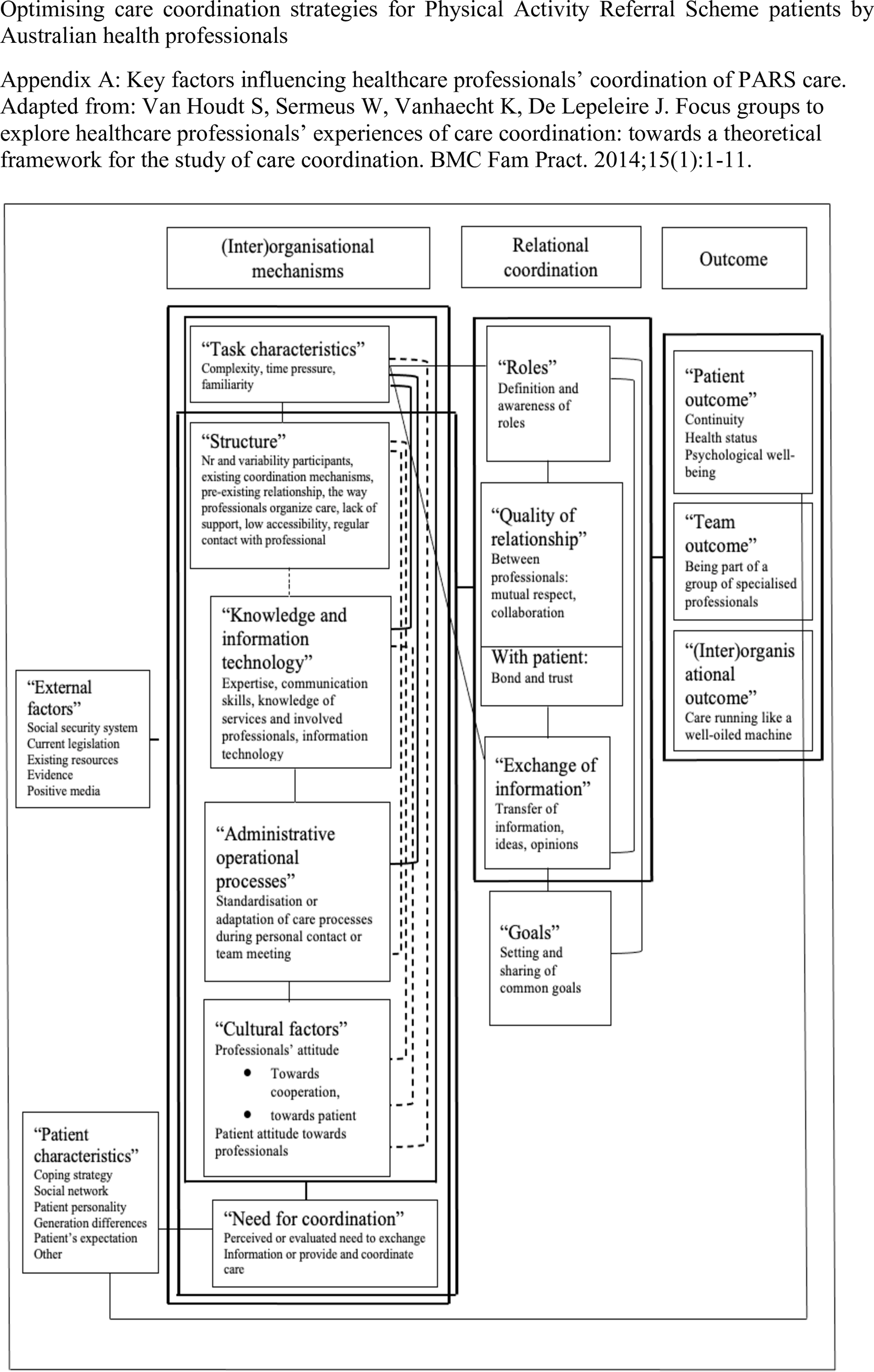

Supplement: S1 Appendix — (TIFF) [file pone.0270408.s001.tiff]
