# Supplementary material for: Optimising care coordination strategies for physical activity referral scheme patients by Australian health professionals
Source: PLoS One. 2022 Jul 14;17(7):e0270408. doi: 10.1371/journal.pone.0270408 (PMC9282539; doi:10.1371/journal.pone.0270408)
Supplement: S2 Appendix — (PDF) [file pone.0270408.s002.pdf]

S2 Appendix B: Interview guide for participants (GPs and exercise physiologist - EPs)

A. GPs’ Interview Guide

No	Questions	Probes and Prompts
A. Role		
1.	To start with, can your briefly explain your role in the management of patients with chronic and lifestyle diseases?	Where is your practice located?<br>Would you consider your practice public or private?<br>What is your level of involvement with patients?<br>What kind of health challenges inform your choice of PARS referral?<br>What was their lifestyle like?<br>Could their lifestyle have predisposed them to lifestyle diseases?
B. PARS knowledge		
2.	Now focusing on the physical activity referral scheme (PARS), what are your experiences with PARS and other pathways for PA interventions?	How did you learn about it?<br>Who/what has motivated you to use this pathway?<br>What type of patient do you refer?<br>Did you have any expectations about the referral scheme, and was this expectation met?
C. Professional Relationship with other HCPs (e.g. EPs) and patients		
3.	How have other HCPs such as EPs influenced your knowledge of physical activity?	Possible areas: prescription, counselling and advice and providing factsheet.
4.	How have you influenced your patient’s uptake of PA and PARS?	Possible areas: improved participation in PA, more understanding of the benefits for PA and can perform PA unsupervised.
5.	Do EPs discuss their roles with you and provide feedback on the outcome of their intervention with the patient you refer to them?	What do you think about the feedback?<br>Is it useful for your practice?<br>Does it inform your future referral choice?
D. Perceived Challenges or Barriers to PARS		
8.	From your viewpoint, what are the challenges associated with the physical activity referral scheme/pathway?	What are your thoughts about Medicare or the chronic disease management (CDM) pathways?<br>Possible areas including cost, distance, support, timings etc.
E. Perception on how to Improve PARS		
9.	From your viewpoint, do you think PARS needs improvement?	if yes, what are your suggestions?
F. On a final note:		
10.	Is there anything else you would like to tell me about your experience of the physical activity referral scheme?	To summarise my understanding of what you said…<br>Is there any information you would like to add, rephrase or remove from all you have said today?
This interview is now over. Thank you for your time and have a great day.		

B. EPs' Interview Guide

No	Questions	Probes and Prompts
A. ROLE		
1.	To start with, can your briefly explain your role in the management of patients with chronic and lifestyle diseases?	• Where is your practice located?<br>• Would you consider your practice public or private?<br>• What is your level of involvement with patients?
B. PARS knowledge		
2.	Now focusing on the physical activity referral scheme (PARS), how are patients referred to you?	• What type of patient do you see?<br>• Any specific type of programme for your clients?<br>• What is the duration of this programme?<br>• On a scale of 1 – 10 with 1 being the least and 10 the most, how would you rate the general success or outcomes of your clients?<br>• Can you describe any scenario where you helped change your client's PA behaviour to achieve a goal?
C. Professional Relationship with other HCPs (e.g. GPs) and patients		
3.	In general terms how do you feel about discussion physical activity with your clients?	• What advice do you give them about PA?<br>• What advice gets your patients moving?<br>• Is this advice feasible?<br>• Are they receptive?
4.	Do you get feedback from patients about the outcome of your intervention with them?	• What do they say?<br>• Are they positive?
5.	Do you often discuss your role with GPs and give feedback to them on the outcome of your intervention with patients?	• Has these influenced the number of referrals you get from GPs?<br>• Any reason why?
6.	What do you think are the benefits of physical activity referral pathways?	
D. Perceived Challenges or Barriers to PARS		
7.	From your viewpoint, what are the challenges associated with the physical activity referral scheme/pathway?	• What are your thoughts about Medicare or the chronic disease management (CDM) pathways?<br>• Possible areas including cost, distance, support, timings etc.
E. Perception on how to Improve PARS		
8.	From your viewpoint, do you think PARS needs improvement?	• if yes, what are your suggestions?
F. On a final note:		
9.	Is there anything else you would like to tell me about your experience of the physical activity referral scheme?	• To summarise my understanding of what you said…<br>• Is there any information you would like to add, rephrase or remove from all you have said today?
This interview is now over. Thank you for your time and have a great day.		
