# Supplementary material for: Optimising care coordination strategies for physical activity referral scheme patients by Australian health professionals
Source: PLoS One. 2022 Jul 14;17(7):e0270408. doi: 10.1371/journal.pone.0270408 (PMC9282539; doi:10.1371/journal.pone.0270408)
Supplement: S3 Appendix — (PDF) [file pone.0270408.s003.pdf]

Optimising care coordination strategies for Physical Activity Referral Scheme patients by Australian health professionals

Appendix B: Protocol for the qualitative phase of this study based on the COREQ checklist.

Adapted from:
Tong A, Sainsbury P, Craig J. Consolidated criteria for reporting qualitative research (COREQ): a 32-item checklist for interviews and focus groups. International Journal for Quality in Health Care. 2007. Volume 19, Number 6: pp. 349 – 357.

No	Item	Description	Reported on page #
**Domain 1: Research Team and Reflexivity**			
	Personal Characteristics		
1.	Interviewer/Facilitator	The primary researcher FAA conducted the interviews	8
2.	Credentials	The interviewer (First Author – **FAA**) holds a BSc and an MSc; Other Authors: **AEOMA**: BSc, MSc, PhD; **MJC**: BSc, GradCert TT, PhD **BSMA**: BSc, MSc, GradCert ULT, GradCert Mgt, PhD;	Not applicable
3.	Occupation	**FAA** is a PhD higher degree by research student at the College of Medicine and Dentistry, James Cook University Townsville, Queensland, Australia<br>**AEOMA** is an Associate Professor in the College of Public Health, Medical and Veterinary Sciences, James Cook University, Townsville, Queensland, Australia<br>**MJC** is an Associate Professor at the Division of Tropical Health and Medicine, James Cook University, Townsville, Queensland, Australia<br>**BSMA** is an Associate Professor at the College of Medicine and Dentistry, James Cook University, Townsville Queensland, Australia	42
4.	Gender	By author: FAA: Male, MJC: Female, AEOMA: Male, BSMA: Female	Not applicable
5.	Experience and Training	All authors have vast experiences in research and published articles on PARS in peer-reviewed journals	Not applicable
	Relationship with Participants		
6.	Relationship Established	The interviewer had no prior relationship with any of the interviewees	Not applicable
7.	Participant Knowledge of the Interviewer	Each interview commenced with a verbal acknowledgement of consent. Participants were informed about the objectives of the study during the introductory stages of the interview	8
8.	Interviewer Characteristics	The interviewer did not report bias of any kind	Not applicable
**Domain 2: Study Design**			
	Theoretical Framework		
9.	Methodological Orientation and Theory	As outlined in the manuscript, framework analysis was employed in the qualitative phase of the study	8

10.	Sampling	Participants were purposively selected for the second phase of the study.	7
11.	Method of Approach	Participants were approached through their email or telephone numbers depending on the contact details they provided	Not applicable
12.	Sample Size	25 participants	8
13.	Non-Participation	Non	
	Setting		
14.	Setting of Data Collection	The interviewer was located in a secured office during the interview. Participants acknowledged the comfort of their location before the interview proceeded	Not applicable
15.	Presence of Non-Participants	No	Not applicable
16.	Description of Sample	To be eligible for this study, participant must be a registered GP or EP, above 18 years of age.	7
	Data Collection		
17.	Interview Guide	Ten (10) semi-structured interview questions were used to explore participants' views about coordinating PARS care for patients who utilized the programme's services. Interview questions examined participants' perception of their roles in coordinating PARS referrals for patients, PARS knowledge, beliefs and attitudes, influences of other HCPs (GPs or EPs), perceived challenges and benefits of PARS and their thoughts on how to improve the effectiveness of the patient care coordination for PARS. Prompts and probes were developed concerning the interview topics, when necessary, to kindle further responses from participants. Telephone interviews lasted between 16 and 50 minutes.	8
18.	Repeat Interviews	Repeat interviews were not required	Not applicable
19.	Audio/Visual Recording	Data was collected using audio recording. Visual recording was not required	8
20.	Field Notes	None	Not applicable
21.	Duration	Each interview lasted between 16 and 50 minutes	8
22.	Data Saturation	Yes – interviews continued until data saturation	7
23.	Transcripts Returned	No, but the research team checked the interview transcripts for accuracy	Not applicable
**Domain 3: Analysis and Findings**			
	Data Analysis		
24	Number of Data Coders	Two (2), researchers (FAA and BSMA) independently coded the data and developed and mapped all themes against those of the care coordination model. A series of consensus meeting between FAA and BSMA facilitated the verification of all the codes generated	9
25	Description of The Coding Tree	Yes, see the qualitative data analysis section of the manuscript	8 - 9
26	Derivation of Themes	All themes were derived inductively from the data.	9

27	Software	QSR international's NVivo version 12 for Mac was used in the management of the qualitative data	8
28	Participant Checking	Yes, the interviewer summarized interview accounts with each participant after the interview	8
	Reporting		
29	Quotation Presented	Sample quotes were presented for each of the theme generated	17 - 25
30	Data and Findings Consistent	Yes, data and findings were consistent.	Not applicable
31	Clarity of Major Themes	Yes, major themes are clear and presented in the body of the manuscript	17 - 25
32	Clarity of Minor Themes	Yes, minor themes are clear and presented in the body of the manuscript	17 - 25
